# Correlation Between Plasma Biomarkers Identified in Normal, SCD, and MCI subjects, with PET Amyloid and Tau Neuroimaging

**DOI:** 10.1002/alz70856_107402

**Published:** 2026-01-09

**Authors:** Allal Boutajangout, Arjun V. Masurkar, Ricardo S. Osorio, Ludovic Debure, Wajiha Ahmed, Mobeena Ghuman, Alok Vedvyas, Jon Links, Haiyun Chen, Louisa Bokacheva, Omonigho M Bubu, Elizabeth Pirraglia, Joshua Chodosh, Brianna Vega, Yongzhao Shao, Karyn Marsh, Henry Rusinek, Thomas Wisniewski

**Affiliations:** ^1^ NYU Alzheimer's Disease Research Center, New York, NY, USA; ^2^ NYU Grossman School of Medicine, New York, NY, USA; ^3^ Center for Cognitive Neurology, New York, NY, USA; ^4^ Center for Cognitive Neurology, New York University Langone Health, New York, NY, USA; ^5^ Center for Sleep and Brain Health, Department of Psychiatry, NYU Langone Health, New York, NY, USA; ^6^ Department of Psychiatry, New York, NY, USA; ^7^ Department of Population Health, New York, NY, USA; ^8^ NYU Grossman School of Medicine, New York, NY, USA; ^9^ New York University Grossman School of Medicine, New York, NY, USA; ^10^ Alzheimer's Disease Research Center, New York University Langone Health, New York, NY, USA

## Abstract

**Background:**

Plasma biomarkers may allow efficient, less invasive methods to assess early‐stage AD/ADRD. Moreover, they can canvas multiple processes, including inflammation, vascular disease, and blood‐brain barrier (BBB) dysfunction. How they reflect cerebral amyloid and tau deposition remains an area of active exploration.

**Method:**

Participants were enrolled at the NYU Alzheimer's Disease Research Center. PET assessment of amyloid pathology was conducted in 163 subjects of whom 62 had normal cognition (NL‐mean age=71.2, 60% female), 82 had subject cognitive decline (SCD‐mean age=76.2, 72% female), and 19 had mild cognitive impairment (MCI‐mean age=73.8, 68% female). Tau PET was conducted in 122 subjects, who were categorized as follows: NL *n* = 44(mean age=71.5, 59% female); SCD *n* = 64 (mean age=76.5, 72% female), and MCI *n* = 14 (mean age=76.8, 71% female). Plasma biomarkers assays were conducted for Aβ40, Aβ42, NfL, GFAP, pTau 181 measured using the SIMOA HD‐X. Plasma biomarkers of neuroinflammation (hIL12p70, hIL1b, hIL4, hIL5, hIFNg, hIL6, hIL8, hIL22, hTNFa, hIL10) and BBB dysfunction (hang2, hHBEGF, hPLGF, hFGFB, hVEGF) was measured with Corplex Cytokine 10‐Plex kit and the angiogenesis 6‐Plex kit, respectively using the Simoa SP‐X.

**Result:**

In linear regression models on *n* = 163 participants, amyloid PET uptake values were regressed on the plasma biomarkers adjusted for age, sex, and cognitive group. Higher amyloid PET uptake was trending with lower plasma Aβ42 (standardized β=‐0.11, 95%CI [‐0.24,0.02], *p* = .09), and associated with higher pTau181 (standardized β=0.19, 95%CI [0.02,0.36], *p* = .03), and higher pTau181/Aβ42 ratio levels (standardized β=0.21, 95%CI[0.06,0.36], *p* = .008). In *n* = 122 participants, tau PET uptake values were regressed on the plasma biomarkers adjusted for age, sex, and cognitive group. Higher tau PET uptake was associated with lower plasma Aβ42 (standardized β=‐0.26, 95%CI [‐0.46,‐0.06], *p* = .013), higher GFAP levels (standardized β=0.20, 95%CI[0.04,0.36], *p* = .015), and lower hVEGF levels (standardized β= 0.27, 95%CI[‐0.45,‐0.08], *p* = .005).

**Conclusion:**

Linear regression with adjustment for age, sex and cognitive group showed a higher amyloid PET uptake associated with high pTau 181, and high pTau 181/Aβ42 ratio. Higher tau PET uptake was associated with lower Aβ42, higher GFAP and lower hVEGF

